# Quality of Life in Patients with Gynecological Cancers: A Web-Based Study

**DOI:** 10.31557/APJCP.2020.21.7.1969

**Published:** 2020-07

**Authors:** Elham Shirali, Fariba Yarandi, Marjan Ghaemi, Ali Montazeri

**Affiliations:** 1 *Yas Hospital, Tehran University of Medical Sciences, Tehran, Iran. *; 2 *Kamali Hospital, Alborz University of Medical Sciences, Karaj, Iran. *; 3 *Health Metric Research Center, Iranian Institute for Health Sciences Research, ACECR, Tehran, Iran. *

**Keywords:** Quality of life, gynecological cancer, women’s health, EORTC QLQ-C30, HADS

## Abstract

**Introduction:**

Gynecological cancers are common in adult women. One of the most important goals in the management of these patients is to improve quality of life, along with survival as a traditional outcome. The aim of this study was to evaluate quality of life in gynecological cancers in Iran.

**Methods:**

This cross-sectional study was performed on a sample of patients with gynecological cancers including uterine, ovarian, cervical, and vulvovaginal attending a teaching hospital affiliated to Tehran University of Medical Sciences between 2014 and 2019. The data was collected by a web-based platform with validated self-administered questionnaires including demographic information, the EORTC QLQ-C30 and the Hospital Anxiety and Depression (HADS). The data were analyzed using appropriate tests.

**Results:**

In all 251 patients were studied. The mean age of patients was 52.8±12.4 years and 43% had uterine, 30% had ovarian, 25% had cervical, and 2% had vulvovaginal cancer. The mean global quality of life score as measured by the EORTC QLQ-C30 was 59.8 ± 24.9. Women with ovarian cancer had the highest and women with cervical cancer had the lowest global quality of life score. There were significant differences in emotional, cognitive and global quality of life by cancer diagnosis (p <0.05). Although not significant, overall physical, role, cognitive and social functioning was found to be better in women who had been treated with surgery. The mean anxiety and depression score were 8.7± 5.0 and 7.1 ± 5.2, respectively.

**Conclusion:**

The results demonstrated that patients with gynecological cancers had a low quality of life, and experience higher anxiety and depression.

## Introduction

Gynecological cancers including cervical, ovarian, uterine and vaginal and vulvar cancer represent around 1 in 5 of all cancers diagnosed in women (Cancer, 2018). However, cervical cancer is more common in premenopausal women, while the incidence of uterine and ovarian cancers increase in the perimenopausal years (Goncalves, 2010) and vaginal and vulvar cancers are uncommon and mostly affect elderly women (Carter and Downs, 2012). Despite the high morbidity and mortality rate of gynecological cancers, cervical and uterine cancers have a high chance of survival (Reis et al., 2010).

Women suffering from gynecological cancer encounter with personal interpretation of cancer, physical impact of the disease, long and transient side effects of the treatment regimens and the reaction of family and friends (Pinar et al., 2008). Indeed, they experience numerous stressors likewise financial difficulties and relationship problems (Golden-Kreutz et al., 2005).

Although during the past decade there have been great advances in the treatment of cancer, treatment strategies still are debilitating patients’ life as they cause decreasing cardio-respiratory capacity, pain, fatigue, and suppressing immune function. In addition, psychological stress, anxiety, depression, fear of recurrence and sleep dysfunction are the other symptoms after cancer treatment that worsen quality of life in these patients (Goncalves, 2010; Lerman et al., 2012). As such some influencing organizations recommended that the goal of treatment of any cancer in addition to improved survival should be improvement in quality of life (Arriba et al., 2010). 

There are a number of studies on quality of life in gynecological cancers. Indeed, it is argued that the disease has both short- and long-term effects on patients’ quality of life. The short-term effects usually are health-related, while log-term effects in addition to general well-being, includes psychosocial and work-related issues. For instance, a recent study on long-term quality of life in women with gynecological cancer reported that the main determinants of poor health related quality of life were comorbidities, deprivation, lack of availability and satisfaction with social support, and psychological outcomes (Mamguem Kamga et al., 2019). Overall studies on quality of life in patients with gynecological cancer are limited (Fang et al., 2015; Afiyanti et al., 2018; Hediya Putri et al., 2018; La Rosa et al., 2019). However, of these a number of papers are reviews (Leppert W, 2015; Chow et al., 2016; Izycki et al., 2016). A recent review of 11 studies involving 975 gynecological cancer patients on psychoeducational interventions to improve sexual functioning, quality of life, and psychological outcomes reported that such interventions could improve depressive symptoms and mental aspect of the quality of life in this population (Chow et al., 2016). Despite these, it seems that more studies are needed to provide sufficient evidence on quality of life in women who suffer from gynecological cancer (Dahl et al., 2013a). Fortunately, recent developments on electronic communications allow collecting such information via web-based platform. As such it is argued that the use of electronic devices could simplify data acquisition, and accelerate information transfer between patients and clinicians (Richter et al., 2008) and is a new area in cancer research (Aktas et al., 2015) which even could decrease patients‘ burden in filling in different questionnaires especially those are long and time consuming.

However, although using computer-based or electronic questioners are becoming popular among investigators, few quality of life studies reported that collected data using web-based technology or electronic devices (Richter et al., 2008; Aktas et al., 2015). The objective of this study was to investigate the quality of life and psychological well being in patients with gynecological cancer using a new mobile device technology.

## Materials and Methods

*Design and patients*

This was a cross-sectional study to assess quality of life using a web-based platform. The study included a sample of women with confirmed diagnosis of gynecological cancers referred to a teaching hospital affiliated to Tehran University of Medical Sciences, Tehran, Iran between 2014 and 2019. The contact information of patients was obtained from electronic health records (EHRs) of the hospital. Eligibility criteria included the following conditions: at least three months from completion of treatment, no recurrence of the disease, ability to understand and communicate in Persian, and having adequate electronic literacy. Patients with psychiatric disorders and severe medical conditions were excluded. The ethics committee of Tehran University of Medical Sciences approved the study.

*Data collection*

**Figure 1:**
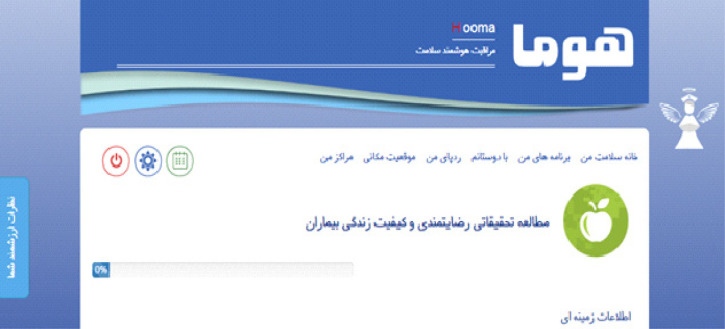
A Screen Shot of the Starting Page of the Web Site

A web-based platform was designed to collect data online. A professional team were designed the platform with multiple layers of security to make sure that data remains private and secure. An expert in health and cancer research made a telephone call to each participant to introduce the study. After asking for consent to participate in the study, a direct link to the questionnaires was sent via SMS (Short Message Service). By clicking on the link, patients were able to enter to the web site under commercial name Hooma to complete the questionnaire. Patients could see the questionnaires on the computer screen while entering their own mobile number as identification code. The questions could be answered via ticks for the appropriate answer. The system was set to notify participants if a question was missed. We indicated that family members could provide assistance. Once completed patients were encouraged to save their responses by clicking on finish bottom. The computer program was designed to allow only one response from any unique phone number and computer IP. Therefore, the patients could complete the forms only once. We contacted any single patients before completing the questionnaire to insure that patients themselves are completing the forms. The data were properly secured when stored on a computer and a password accessed server. Data was collected as a spreadsheet and remained anonymous with no information linking questionnaires to participants. Figure 1 presents a screen shot of the starting page (https://www.hooma.salemsa.net).

*Questionnaires*

1. Demographic and clinical information: This included item on age, education, occupation, and income, time since diagnosis and information about the type of cancer, treatment modalities and date of their last treatment.

2. Quality of life: The European Organization for Research and Treatment of Cancer (EORTC) core quality of life questionnaire (QLQ-C30) was used to assess quality of life. The EORTC-QLQ-C30 is a 30-item self-reported instrument measuring physical (5 items), emotional (4 items), role (2 items), cognitive (2 items) and social (2 items) functioning as well as global health status (2 items). Higher scores on these scales represented better functioning. The questionnaire also contains items measuring nausea and vomiting (2 items), fatigue (3 items) and pain (2 items), and 6 single questions measuring dyspnea, insomnia, appetite loss, constipation, diarrhea and financial impact. Higher scores for symptoms indicate greater symptoms. The scores on all scales range from 0 to 100 (Osoba et al., 1997). We used the Iranian version of the questionnaire. The psychometric properties of the Iranian version are well document (Montazeri et al., 1999).

3. Psychological distress: This was assessed using the Hospital Anxiety and Depression Scale (HADS). It is a self-assessment instrument with 14 items and two separate subscales for anxiety and depression; seven items for the anxiety subscale (HADS Anxiety) and seven items for the depression subscale (HADS Depression). Each item is rated on a four-point Likert scale ranging from0 and 3 giving a total score of 0 to 21 for each subscale. The higher score on either subscale indicates worse situation. Recommended cut-off scores for the questionnaire reads as follows: 0-7 (normal), 8–10 (borderline) and ≥11 considers as case (Zigmond and Snaith, 1983). We used the Iranian version of the questionnaire. The psychometric properties of the questionnaire are well documented (Montazeri, 2003).

*Data analysis*

Descriptive analysis was used to explore the data. Qualitative variables are presented as number and percentage, and quantitative variables are presented as mean (SD). QoL scores were described and compared across cancer type. Continuous variables were compared using the one-way analysis of variance (ANOVA) with post hoc analysis, and categorical variables were examined using Pearson’s chi-square test with continuity correction. A two-sided p<0.05 was considered statistically significant. Data were analyzed with SPSS 24 for Windows (SPSS Inc, Chicago, Illinois).

## Results

*Demographic and pathological characteristics of the patients*

**Figure 2:**
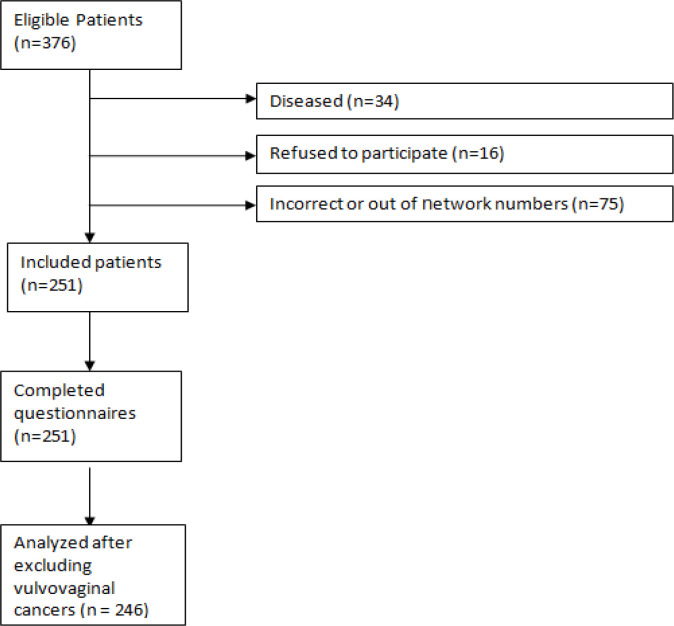
Flow Chart of the Study Participants. Number of women who passed away or refused to participate and the phone numbers those were incorrect or out of network

In all of 376 eligible patients, 251 patients with confirmed diagnosis of gynecological cancer were entered into the study and completed the web-based questionnaires (Figure 2). None of the participants refused to participate in the study due to lack of Internet access. All patients completed the questionnaires successfully. However, since the number of patients with vulvovaginal cancer was limited (n = 5) these patients were excluded from further analysis (n = 246). The mean (SD) age of patients was 52.8 (12.4) years ranging from 27 to 80. About 85% were housewives. The time since diagnosis in most patients was between 1 to 5 years. The demographic characteristics of the patients are presented in Table 1.

*Quality of life*

Functioning: In general women with cancer of cervix reported lower quality of life compared to women with cancer of ovary and uterus. There were significant differences among the study groups in terms of emotional functioning (P = 0.033), cognitive functioning (P = 0.012) and global quality of life (P = 0.042). The lowest and highest scores were for global quality of life and physical functioning respectively (Table 2).

Symptoms: Although women with cervical cancer reported higher symptoms compared to patients with uterus and cervix cancers, there were no significant differences among the study groups except for fatigue (P = 0.030) and financial difficulties (P = 0.046). The scoring patterns among patients with ovarian and cervical cancers were very similar (Table 2). 

Treatment modalities: The EORTC QLQ-C30 scores based on treatment modalities adjusting for age modification are summarized in Table 3. As shown the only significant difference among patients who received different treatment was diarrhea (P=0.048).

*Anxiety and depression*

There were no significant differences in anxiety and depression among the study groups after adjusting the scores for age. However, patients with cervical cancer scored higher on both anxiety and depression subscales. The results are shown in Table 4.

**Table 1:** Basic Demographics and Treatment Characteristics of the Patients

	Total (n=246)	Uterus (n=109)	Ovary (n=74)	Cervix (n=63)	P*
Age (years)					
Mean (SD)	52.8(12.4)	55.1(10.1)	47.6(14.7)	53.0(13.8)	<0.001
Education (%)					0.142
Illiterate	15.4	15.6	10.8	20.6	
Primary	25.2	31.2	20.3	20.6	
Secondary	41.5	41.3	45.9	36.5	
Higher	17.9	11.9	23	22.2	
Working Status (%)					0.12
Working	14.2	9.2	17.6	14.2	
Housewife	85.8	90.8	81	85.8	
Income (%)					0.871
Poor/fair	55.3	60.6	44.6	58.7	
Good	44.7	39.4	55.4	41.3	
Time since diagnosis (years) (%)					0.255
<1 year	17.4	21.3	18.8	9.1	
Between 1 to 5 years	78.4	73.4	76.6	89.1	
> 5 years	4.2	5.3	4.7	1.8	
Treatment**					< 0.001
Surgery	79	38	33	8	
Chemoradiotherapy	66	24	1	41	
Surgery & Chemotherapy	45	10	34	1	
Surgery & Chemoradiotherapy	40	25	4	11	
Surgery & Radiotherapy	16	12	2	2	

*, P-values derived from one-way analysis of variance for continuous variables, and Pearson's chi-square test for categorical variables; **, Derived from analysis of covariance (age as covariate)

**Table 2:** Quality of Life Scores as Measured by the EORTC QLQ-C30 by Cancer Diagnosis

	Uterus (n=109)	Ovary (n=74)	Cervix (n=63)	P***
	Mean (SD)	Mean (SD)	Mean (SD)	
Functioning*				
Physical	75.9 (2.3)	79.8 (9.4)	77.6 (5.0)	0.665
Role	83.5 (2.7)	90.6 (11.2)	86.4 (5.9)	0.282
Emotional	66.1 (3.0)	52.1 (12.3)	51.1 (6.5)	0.625
Cognitive	84.9 (2.2)	87.3 (9.3)	72.2 (4.9)	0.773
Social	85.0 (2.8)	86.5 (11.6)	77.4 (6.1)	0.162
Global quality of life	61.8 (2.7)	61.8 (11.0)	51.6 (5.8)	0.856
Symptoms**				
Fatigue	29.6 (2.9)	19.8 (11.9)	36.7 (6.3)	0.407
Nausea and Vomiting	7.6 (1.8)	5.1 (7.5)	8.2 (4.0)	0.964
Pain	24.3 (2.9)	17.2 (12.0)	26.0 (6.4)	0.611
Dyspnea	13.1 (2.5)	15.0 (10.2)	17.8 (5.4)	0.338
Insomnia	35.1 (3.7)	30.4 (15.0)	36.2 (7.9)	0.945
Appetite Loss	14.6 (3.1)	12.9 (12.5)	9.6 (6.6)	453
Constipation	21.0 (3.0)	15.9 (12.3)	27.0 (6.5)	0.506
Diarrhea	7.5 (1.8)	1.8 (7.5)	3.6 (4.0)	0.048
Financial Difficulties	32.0 (4.1)	33.6 (16.6)	54.4 (8.8)	0.435

*, Higher scores in Global Health Status and Functional Scale indicate better quality of life; **, Higher scores in Symptom Scales, indicate worse quality of life; ***, Derived from analysis of covariance (age as covariate).

**Table 3:** The EORTC QLQ-C30 Module Scores Based on Treatment Modalities Adjusting for Age

	Surgery	Chemoradiotherapy	Surgery & Chemotherapy	Surgery & Chemoradiotherapy	Surgery & Radiotherapy	P***
	Mean (SD)	Mean (SD)	Mean (SD)	Mean (SD)	Mean (SD)	
Functioning*						
Physical	84.2 (2.9)	79.9 (14.5)	74.7 (5.8)	66.3 (4.5)	83.8 (7.1)	0.742
Role	90.1 (3.5)	96.2 (17.3)	83.6 (6.9)	73.6 (5.4)	90.6 (8.5)	0.957
Emotional	63.7 (3.8)	48.7 (19.0)	51.0 (7.6)	52.5 (6.0)	66.4 (9.4)	0.161
Cognitive	86.2 (2.9)	87.2 (14.3)	69.2 (5.7)	75.2 (4.5)	89.5 (7.0)	0.686
Social	89.8 (3.6)	89.3 (17.8)	65.1 (7.2)	73.9 (5.6)	96.7 (8.8)	0.24
Global quality of life	64.1 (3.4)	53.7 (16.9)	56.0 (6.8)	50.2 (5.3)	67.8 (6.3)	0.905
Symptom Scales**						
Fatigue	27.1 (3.7)	5.7 (18.3)	39.3 (7.6)	49.0 (5.7)	22.4 (9.0)	0.205
Nausea and Vomiting	5.8 (2.3)	1.5 (11.6)	15.0 (4.7)	11.6 (3.6)	0.9 (5.7)	0.728
Pain	15.6 (3.7)	10.2 (18.5)	30.3 (7.4)	40.6 (5.8)	15.7 (9.1)	0.689
Dyspnea	16.3 (3.2)	17.6 (15.7)	21.4 (6.3)	9.3 4.9)	11.9 (7.7)	0.38
Insomnia	28.5 (4.7)	32.3 23.0)	39.4 (9.3)	39.1 (7.2)	30.2 (11.4)	0.493
Appetite Loss	7.5 (3.9)	2.1 (19.3)	16.4 (7.7)	29.6 (6.0)	10.5 (9.5)	0.565
Constipation	21.3 (3.8)	6.3 (19.0)	43.8 (7.5)	16.8 (5.9)	18.2 (9.3)	0.353
Diarrhea	3.6 (2.3)	3.8 (11.6)	2.4 (4.6)	6.3 (3.6)	0.83 (5.7)	0.343
Financial Difficulties	28.4 (5.2)	28.3 (25.6)	57.6 (10.3)	51.1 (8.0)	34.6 (12.6)	0.115

*, Higher scores in Global Health Status and Functional Scale indicate better quality of life; **, Higher scores in Symptom Scales, indicate worse quality of life; ***, Derived from analysis of covariance (age as covariate)

**Table 4:** Anxiety and Depression by Cancer Site

	Uterus (n=109)	Ovary (n=74)	Cervix (n=63)	
	Mean (SD)	Mean (SD)	Mean (SD)	P**
Anxiety*	7.9 (5.0)	8.8 (5.0)	9.8 (5.0)	<0.001
Depression*	6.4 (5.2)	7.2 (5.2)	8.0 (5.0)	<0.001

*, Higher scores indicate higher levels of anxiety and depression; **, Derived from analysis of covariance adjusted for age

## Discussion

Quality of life impairment is an important outcome measure among cancer patients. This study reported on quality of life among women with gynecological cancer via online method by using a well-known questionnaire. Electronic data collection may represent the patient history and also give a comprehensive picture of the population health status (Gentil et al., 2017). They provide opportunities to enhance patient care, embed performance measures in clinical practice, and facilitate clinical research (Cowie et al., 2017). This method is also acceptable in studies related to health quality of life in cancers (Matthew et al., 2007; Kikawa et al., 2019). The EORTC Quality of Life Group has recently developed computerized adaptive tests (CATs) for the EORTC QLQ-C30 quality of life questionnaire that may be usable for general applications (Petersen et al., 2020). Studies have shown that computer QOL questionnaires are well accepted by cancer patients, with good data quality and reliability (Velikova et al., 1999) and administration of such questionnaires in clinical and medical oncology inpatient practice is feasible (Buxton et al., 1998). A study reported that the percentage of patients who agreed to take part in online study was comparable with the rate of compliance with QOL studies in therapeutic clinical trials (Velikova et al., 1999). Indeed, using computerized adaptive tests may improve precision and smaller sample requirements, compared to the static QLQ-C30 (Petersen et al., 2020). 

We evaluated QoL based on tumor site and treatment modalities and found that women with gynecological cancer suffer from a relatively poor quality of life. Modern management of cancer in addition to patients’ management includes psychological and social aspects in order to achieve a better QoL (Reis et al., 2010). Pearman et al. found that QoL of gynecological cancer patients was most negatively affected around time of diagnosis and treatment. At 6 to 12 months after treatment, there was no difference in overall QoL compared with age-matched controls (Pearman, 2003).

The statistical evaluation in the study revealed that the type of cancer had a major influence on the patient’s QoL. Women with ovarian or endometrial cancer had a better health status, role functioning and social well-being than those with cervical cancer. These may be due to various treatment modalities needed in cervical cancer. The chemotherapy and specifically the radiation received by these women can lead to developing symptoms such as sexual dysfunction and urinary and bowel dysfunction that perhaps affect women in unique ways.

We found that emotional score was the lowest functioning score. In addition, we found that the second most affected parameter was physical well-being. Physical problems may arise in the post-treatment period, while exhaustion, as one of these problems, had a major effect on the physical functions (Reis et al., 2010). Similar finding was reported by other investigators where physical role was the worse score in scaling (Özaras G, 2010). Social aspect was good in our patient. This might be due to high familial support in our culture. 

Fatigue is the most significant problem affecting the daily activities and life of cancer patients (Hoskins NS, 1997). Similarly in the present study, fatigue score was found to be the highest symptom score. The second and third highest scores were insomnia and pain respectively.

It seems that psychosocial status at time of diagnosis is determining QoL and well-being in the long term. Association has been found between risk of depression and anxiety in the long term after cancer (Dahl et al., 2013b). In our study, the score of anxiety and depression were at borderline; which means that patients were prone to mood disorder. Although not statistically significant, it was worse in cervical cancer. Indeed, Osann et al. (2014) reported a high level of depression 9 to 30 months after diagnosis of cervical cancer. It is important that social support should be given to the patients to reduce anxiety. It may be useful to help patients to cope with the disease process and achieve better QoL.

Considering lower QoL in gynecological cancer, it may be crucial to improve it. One of the concerns in these patients is fear of recurrence (Hodgkinson et al., 2007) Available findings are crucial to develop interventions to support those at risk for QoL impairments. Future research efforts should identify not only how these will affect QoL but also develop strategies for identifying women at risk of serious QoL disruption. Efforts should also be focused on developing effective interventions to prevent or minimize the detrimental effects of both gynecological cancer and treatment on the QoL of patients and to identify the specific QoL needs of the patients.

Rehabilitation centers and psychosocial approaches to the cancer patients may have a positive effect in the therapy and prognosis of these patients. Health care providers have important role in providing social support to the patients and to their families, and gynecologist and nurses have a characteristic role in establishing the positive interaction between patients and their relatives.

About 67% of our expected patients participated in the study. We could not reach most of them due to mortality or failure to reach their phone number. However, all participants completed the questionnaire after first call or one reminder call.

The strengths of our study are the administration of validated instruments to assess quality of life and psychological features. Studies on quality of life in gynecological cancer are few (Montazeri et al., 1996). Indeed, we successfully used a web-based platform to collect data that could help us to re-evaluate the patients for long term and interventional studies. The high completion rate in our study showed that electronic assessment is feasible. Upon completion of the assessment, the rapid transfer of results helped integrate systematic symptom assessment into the daily report in an oncology clinic as a study also showed similar benefits (Bennett et al., 2012). The limitation of our study was small sample size, which represented data only from patients of a single hospital. Patients in this study were not representative of elderly cancer patients and thus the results might not be generalized due to the lower Internet literacy among elderly patients..

In conclusion, the findings suggest that quality of life among patients with gynecological cancer is low and especially they experience poor emotional and physical functioning and are susceptible for mood disorder. Indeed, to ensure the continuity of quality care, measuring quality of life in patients with gynecological is essential.
